# Institutional Needs

**Published:** 1913-04-12

**Authors:** 


### Institutional Needs.

### "MELDRUM" REFUSE DESTRUCTORS.

It is now recognised that asepsis in the ward or theatre is of little avail if external centres of infection are permitted to exist; and that flies in the ward, for instance, are best banished not by traps, but by the destruction of their breeding-places, of which refuse is perhaps the chief. "Meldrum" Patent Refuse Destructors are noteworthy, being small, high-temperature destructors, with a burning capacity ranging from 25 lb. per hour to half a ton or more. So many designs have been made for special purposes and for all sizes and types of institutions that detailed specifications should be made to the firm at Timperley, near Manchester. Forced draught can, of course, be supplied, and where used enables coke, cinders, and good class refuse to be used for starting up. The plants are so constructed that no offensive or dangerous gases can escape unburnt. A high and uniform temperature is maintained in the combustion chamber, ensuring the thorough cremation of all fumes and vapours. For very moist or semi-liquid material, a dished firebrick hearth is provided at the front of the cell. The moisture is here driven off (the fumes having to pass over the incandescent fire beyond), and when dried the refuse is pushed forward on to the grate, where it readily ignites.

### GOLD MEDAL FOR SULPHO-"VASELINE."

Last year Sulpho-"Vaseline" for dogs was awarded gold medal and highest award at Cruft's Great International Dog Show at the Royal Agricultural Hall, Islington. Supreme honours have again gone to the Chesebrough Manufacturing Company (Consolidated) for their Dog-"Vaseline" Exhibit at the recent Cruft's Show held at the same place in February last. This product is, of course, an entirely separate and distinct speciality from those advertised and sold for human needs, just as it differs from the Veterinary-"Vaseline" supplied for horses and cattle.

